# Glioblastoma in the real-world setting: patterns of care and outcome in the Austrian population

**DOI:** 10.1007/s11060-024-04808-x

**Published:** 2024-08-27

**Authors:** Andreas Hainfellner, Martin Borkovec, Lukas Seebrecht, Magdalena Neuhauser, Thomas Roetzer-Pejrimovsky, Lisa Greutter, Birgit Surböck, Andrea Hager-Seifert, Doris Gorka-vom Hof, Tadeja Urbanic-Purkart, Martin Stultschnig, Clemens Cijan, Franz Würtz, Bernadette Calabek-Wohinz, Josef Pichler, Isolde Höllmüller, Annette Leibetseder, Serge Weis, Waltraud Kleindienst, Michael Seiberl, Lara Bieler, Constantin Hecker, Christoph Schwartz, Sarah Iglseder, Johanna Heugenhauser, Martha Nowosielski, Claudius Thomé, Patrizia Moser, Markus Hoffermann, Karin Loibnegger, Karin Dieckmann, Matthias Tomschik, Georg Widhalm, Karl Rössler, Christine Marosi, Adelheid Wöhrer, Johannes A. Hainfellner, Stefan Oberndorfer

**Affiliations:** 1 Division of Neuropathology and Neurochemistry, Department of Neurology, Comprehensive Center for Clinical Neurosciences and Mental Health, Medical University of Vienna, Medical University Campus AKH 4J, Waehringer Guertel 18-20, 1090 Vienna, Austria; 2 Division of Anatomy, Center for Anatomy and Cell Biology, Medical University of Vienna, Vienna, Austria; 3 Center for Medical Physics and Biomedical Engineering, Medical University of Vienna, Vienna, Austria; 4 Department of Neurology, Clinic Favoriten, Vienna, Austria; 5 Department of Neurology, State Hospital Wr. Neustadt, Wr. Neustadt, Austria; 6 Department of Neurology, Medical University of Graz, Graz, Austria; 7 Department of Neurology, State Hospital Klagenfurt, Klagenfurt, Austria; 8 Department of Pathology, State Hospital Klagenfurt, Klagenfurt, Austria; 9 Department of Neurology, University Hospital St. Pölten, Dunant-Platz 1, 3100 St. Pölten, Austria; 10 Department of Internal Medicine and Neuro-Oncology, Neuromed Campus, Kepler University Hospital, Johannes Kepler University of Linz, Linz, Austria; 11 Department of Neurology, Neuromed Campus, Kepler University Hospital, Johannes Kepler University of Linz, Linz, Austria; 12 Division of Neuropathology, Department of Pathology and Molecular Pathology, Neuromed Campus, Kepler University Hospital, and Clinical Research Institute for Neurosciences, Johannes Kepler University of Linz, Linz, Austria; 13 Department of Neurology, University Hospital Salzburg, Paracelsus Medical University Salzburg, Salzburg, Austria; 14 Department of Neurosurgery, University Hospital Salzburg, Paracelsus Medical University Salzburg, Salzburg, Austria; 15 Department of Neurology, Medical University of Innsbruck, Innsbruck, Austria; 16 Department of Neurosurgery, Medical University of Innsbruck, Innsbruck, Austria; 17 Laboratory of Neuropathology, Tirol Kliniken GmbH, Innsbruck, Austria; 18 Department of Neurosurgery, State Hospital Feldkirch, Feldkirch, Austria; 19 Department of Radiation Oncology, State Hospital Feldkirch, Feldkirch, Austria; 20 Department of Radiation Oncology, Comprehensive Cancer Center, Medical University of Vienna, Vienna, Austria; 21 Department of Neurosurgery, Comprehensive Center for Clinical Neurosciences and Mental Health, Medical University of Vienna, Vienna, Austria; 22 Division of Palliative Care, Department of Internal Medicine I, Comprehensive Cancer Center, Medical University of Vienna, Vienna, Austria; 23 Division of Oncology, Department of Internal Medicine I, Comprehensive Cancer Center, Medical University of Vienna, Vienna, Austria; 24 Karl Landsteiner Institute for Clinical Neurology and Neuropsychology, Department of Neurology, University Hospital St. Pölten, St. Pölten, Austria

**Keywords:** Glioblastoma, Prognosis, Outcome, Registry

## Abstract

**Purpose:**

We present results of a retrospective population-based investigation of patterns of care and outcome of glioblastoma patients in Austria.

**Patients and methods:**

In this nation-wide cooperative project, all Austrian glioblastoma patients newly diagnosed between 2014 and 2018 and registered in the ABTR-SANOnet database were included. Histological typing used criteria of the WHO classification of CNS tumors, 4th edition 2016. Patterns of care were assessed, and all patients were followed until the end of 2019.

**Results:**

1,420 adult glioblastoma cases were identified. 813 (57.3%) patients were male and 607 (42.7%) female. Median age at diagnosis was 64 years (range: 18–88). Median overall survival (OS) was 11.6 months in the total cohort and 10.9 months in patients with proven IDH-wildtype. Median OS in the patient group ≤ 65 years receiving postoperative standard of care therapy was 16.1 months. In the patient group > 65 years with postoperative therapy, median OS was 11.2 months. Follow-up ≥ 5 years identified 13/264 (4.9%) long-term survivors. Brain tumor surgery frequently was assisted by 5-aminolevulinic acid (5-ALA) fluorescence (up to 55%). Postoperative treatment was initiated around one month after surgery (median: 31 days) following standardized protocols in 1,041/1,420 (73.3%) cases. In 830 patients (58.5%), concomitant radiochemotherapy was started according to the established standard of care. Treatment in case of progressive disease was considerably variable. 170/1,420 patients (12.0%) underwent a second surgical procedure, 467 (33.0%) received systemic treatment after progression, and 173 (12.2%) were re-irradiated.

**Conclusion:**

Our data illustrate and confirm nation-wide translation of effective standard of care to Austrian glioblastoma patients in the recent past. In the case of progressive disease, highly variable therapeutic approaches were used, most frequently accompanied by anti-angiogenic therapy. Long-term survival was observed in a minor proportion of mostly younger patients who typically had gross total tumor resection, a favorable postoperative ECOG score, and standard of care therapy.

## Introduction

Glioblastoma is the most common malignant primary brain tumor in adults. [1] Despite its relatively low overall incidence rate of approximately 2–6/100,000 person-years in Western countries [2], glioblastoma causes a high burden of disease and is still fatal. [3] Since the definition of the current standard of care (SOC) in 2005 [4], only two trials reported some prolongation of median overall survival by optimizing diagnostic and therapeutic management [5, 6]. How these effects translate into the real-world and general population settings has only been investigated to a limited extent. For such investigations, population-based brain tumor registries are required, of which some examples already exist, including: Central Brain Tumor Registry of the United States (CBTRUS) [7], French Brain Tumor DataBase (FBTDB) [8], Brain Tumor Registry of Japan (BTRJ) [9], Swedish National Quality Registry for Primary Brain Tumors (SQRBT) [10], Netherlands Cancer Registry (NCR) [11], and the Zurich Glioblastoma Cohort [12].

The Austrian Brain Tumor Registry (ABTR) was implemented as a nationwide database for malignant and non-malignant brain tumors in the Austrian population [13]. A specific focus on glioblastoma was set in a joint effort by the ABTR and the Society of Austrian Neuro-Oncology (SANO), resulting in the ABTR-SANOnet database. This registry was established in 2014 and provides information on patterns of care and outcomes of Austrian glioblastoma patients, in addition to basic demographic data [14, 15]. The purpose of the ABTR-SANOnet cooperation is to attain a greater knowledge of the real-world management approaches and outcomes in Austrian glioblastoma patients. Its goal, besides population-based data, lies in benchmarking and quality control, investigating the translation of treatment standards defined by clinical trials into the general Austrian population. Output of the ABTR-SANOnet is supposed to facilitate exchange within centers across Austria and shall build a basis for international comparisons with patient cohorts and registries of other countries.

The results of the first years since beginning of the ABTR-SANOnet project are presented in this study. As one of the few population-based descriptions of patterns of care and outcomes of glioblastoma patients in the real-world setting of a Western country, it aims at putting the population-based findings in context with existing knowledge, in particular with data from clinical trials.

## Patients and methods

### Ethical approval and data collection

Ethical approval for the study was obtained from the ethics committee of the Medical University of Vienna (ECS 1140/2018).

Data collection was performed using the web-based ABTR-SANOnet database (FileMaker® programming) in cooperation with all Austrian neuro-oncological centers.

**Fig. 1:**
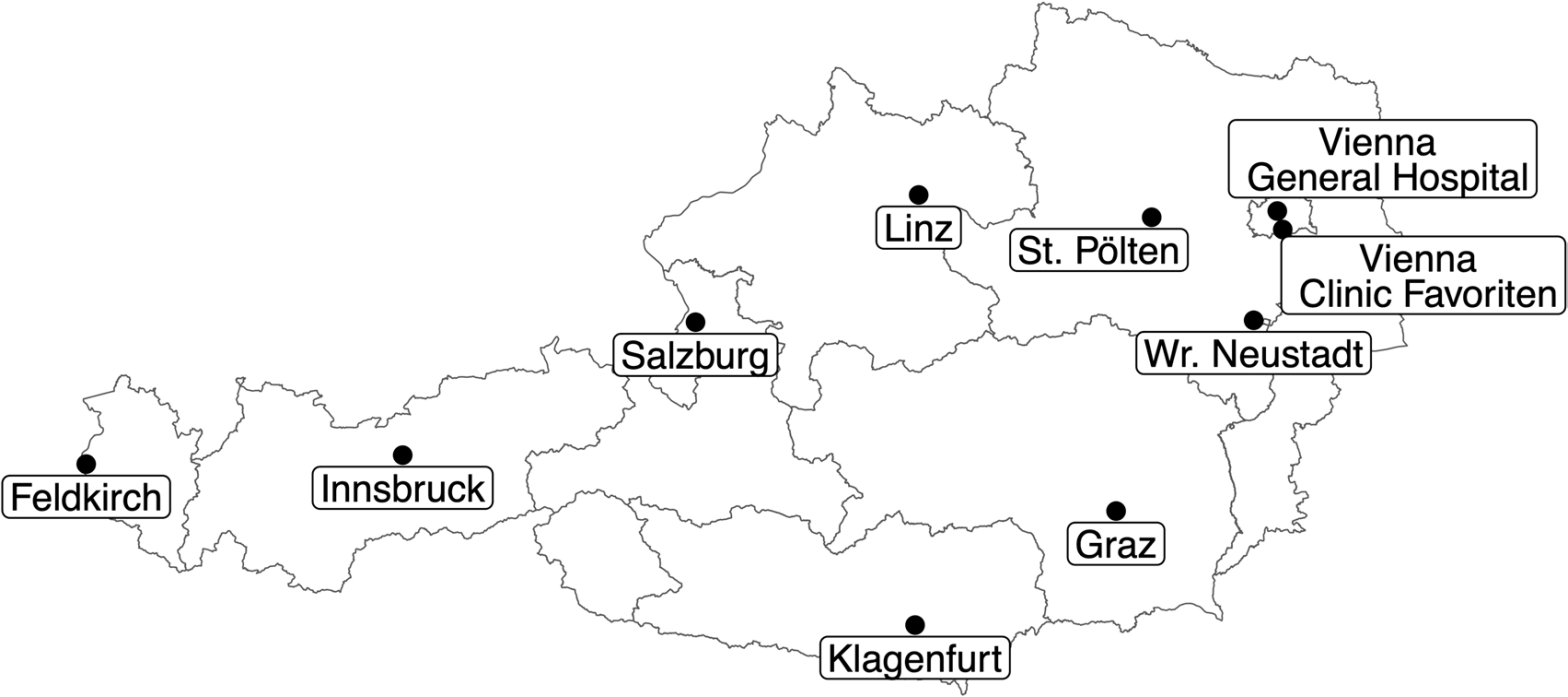
Geographical distribution of cooperating centers across Austria. Data underlying this study stems from all Austrian neuro-oncological centers, which are indicated in their geographical localization on this map of Austria. Names of centers (in alphabetical order): Feldkirch, Graz, Innsbruck, Klagenfurt, Linz, Salzburg, St. Pölten, Vienna Clinic Favoriten, Vienna General Hospital, Wr. Neustadt

Figure 1 shows the distribution of cooperating centers across Austria (in alphabetical order): Feldkirch (State Hospital), Graz (University Hospital and Medical University), Innsbruck (University Hospital and Medical University), Klagenfurt (State Hospital), Linz (Kepler University Hospital and Johannes Kepler University), Salzburg (University Hospital Salzburg and Paracelsus Medical University), St. Pölten (University Hospital), Vienna Clinic Favoriten, Vienna General Hospital and Medical University of Vienna, and Wr. Neustadt (State Hospital).

Patient data was collected following the natural course of glioblastoma management in real life: presenting symptom, first diagnostic scan, first surgical intervention, neuropathological diagnosis, first-line radiotherapy and/or chemotherapy, potential adverse events, second-, third- or multiple-lines therapy including re-resection.

### Inclusion criteria

All adult patients (≥ 18 years) diagnosed histologically between 01.01.2014 and 31.12.2018 with glioblastoma (WHO grade IV) and gliosarcoma (WHO grade IV), based on the World Health Organization (WHO) classification of tumors of the central nervous system, 4th updated edition 2016 [16] in Austria, were included. They were actively followed until 31.12.2019. A permanent address in Austria was required for survival updates by the governmental Austrian National Cancer Registry at Statistics Austria.

### Data grouping and data analysis

Presenting symptoms were clustered into the following categories: focal neurological deficits, headache, epilepsy, personality change, and others. The first diagnostic scan was defined as the first diagnostic magnetic resonance imaging (MRI) showing radiological characteristics of glioblastoma. First surgical interventions were categorized into gross total resection (GTR), subtotal resection (STR), or biopsy, with or without 5-aminolevulinic acid (5-ALA) administration for intraoperative brain tumor visualization [17]. GTR was defined as resection of all contrast enhancing tumor visualized on MRI performed within 48 h after surgery [18]. Patients with maximal safe resection but remaining tumor tissue were assigned to the category STR. A biopsy in this study was defined as removal of a small amount of tumor tissue for diagnostic purposes, therefore including stereotactic and open biopsy procedures. Information on extent of resection was extracted from patient charts.

Data analysis was performed using R statistical software (Version 4.1.1, R Foundation for Statistical Computing, Vienna, Austria) and SPSS® Statistics (IBM®, Version 28.0). Statistical methods included descriptive statistics, survival analyses (Kaplan–Meier, Cox proportional hazards model), and graphical methods such as forest plots. A p-value of < 0.05 was considered statistically significant.

### Neuropathology

Neuropathological tumor typing was performed according to the WHO classification of tumors of the central nervous system 2016 [16] criteria, which included also high-grade IDH-mutant astrocytic gliomas with necrosis and/or vascular proliferation. The fifth most recent edition of the WHO classification was published in 2021 [19] and was implemented across neuro-oncological centers worldwide after completion of data collection. In the present study, the analyzed molecular neuropathological parameters were *IDH1/2* status (wildtype or mutant, detected either by immunohistochemistry (IHC) or DNA sequencing methods) [20] and MGMT promoter methylation status (methylated or unmethylated, by pyrosequencing or methylation-specific PCR (MSP)) [21, 22]. In IDH-mutant tumors, high grade oligodendroglioma was excluded on basis of the 1p/19q status and/or the immunohistochemical ATRX and p53 expression status [16].

### Therapy

Information on parameters associated with first-, second-, multiple-lines therapy, radio- and/or chemotherapy is provided as outlined in the results.

## Results

### Baseline characteristics of study cohort

1,420 adult glioblastoma cases met the inclusion criteria. Table 1 provides a summary of basic clinical characteristics of the patient cohort, of IDH- and MGMT promoter status, and of overall survival.

**Table 1:** Baseline characteristics of study cohort

Characteristic	Subcategory	Number (percent) Total N = 1420 patients
Age (year)		
	Median	64
	Range	18–88
Age groups		
	< 65 year	738 (52.0)
	≥ 65 year	682 (48.0)
Sex		
	Female	607 (42.7)
	Male	813 (57.3)
Presenting symptom		
	Focal neurological deficits	599 (42.2)
	Headache	245 (17.3)
	Epilepsy	221 (15.6)
	Personality change	156 (10.9)
	Other presenting symptoms	162 (11.4)
	Unknown	37 (2.6)
Extent of neurosurgery		
	Total resection	575 (40.5)
	Subtotal resection	393 (27.7)
	Biopsy	430 (30.3)
	Unknown	22 (1.5)
Postoperative ECOG performance status		
	0	389 (27.4)
	1	486 (34.2)
	2	246 (17.3)
	3	97 (6.8)
	4	17 (1.2)
	Unknown	185 (13.0)
IDH-status		
	Wildtype	1115 (78.5)
	Mutated	49 (3.5)
	Unknown	256 (18.0)
MGMT-promoter status		
	Methylated	487 (34.3)
	Unmethylated	504 (35.5)
	Unknown	429 (30.2)
Overall survival (mo)		
	Median	11.6
	Minimum	< 0.1
	Maximum	70.8 (5.9 years)*

*The maximum observation period in this study was 6 years

813 (57.3%) patients were male (M) and 607 (42.7%) female (F), the M/F-ratio was 1.3. Median age at diagnosis was 64 years (range: 18–88 years).

IDH-status was wildtype in 1,115/1,420 (78.5%), IDH-mutation was detected in 49 (3.5%), and in 256 (18.0%) IDH-status was unknown (either not analyzed, or result was not available during data collection). MGMT-promoter was methylated in 487 (34.3%), whereas 504 (35.5%) showed no MGMT-promoter-methylation. MGMT-status was unknown in 429 (30.2%).

### Clinical characteristics

Most patients presented with focal neurological deficits (599, 42.2%), followed by headache (245, 17.3%), epilepsy (221, 15.6%), personality changes (156, 10.9%), and other presenting symptoms (162, 11.4%; in 37 cases (2.6%), presenting symptoms were unknown). Other presenting symptoms/reasons for patient presentation included: incidental detection of glioblastoma during hospitalization, or as part of the staging of preexisting malignancies; emergency indication of resection for decompression (to reduce intracranial hypertension, e.g., due to an acute intracerebral hemorrhage), and subsequent histological diagnosis.

**Fig. 2:**
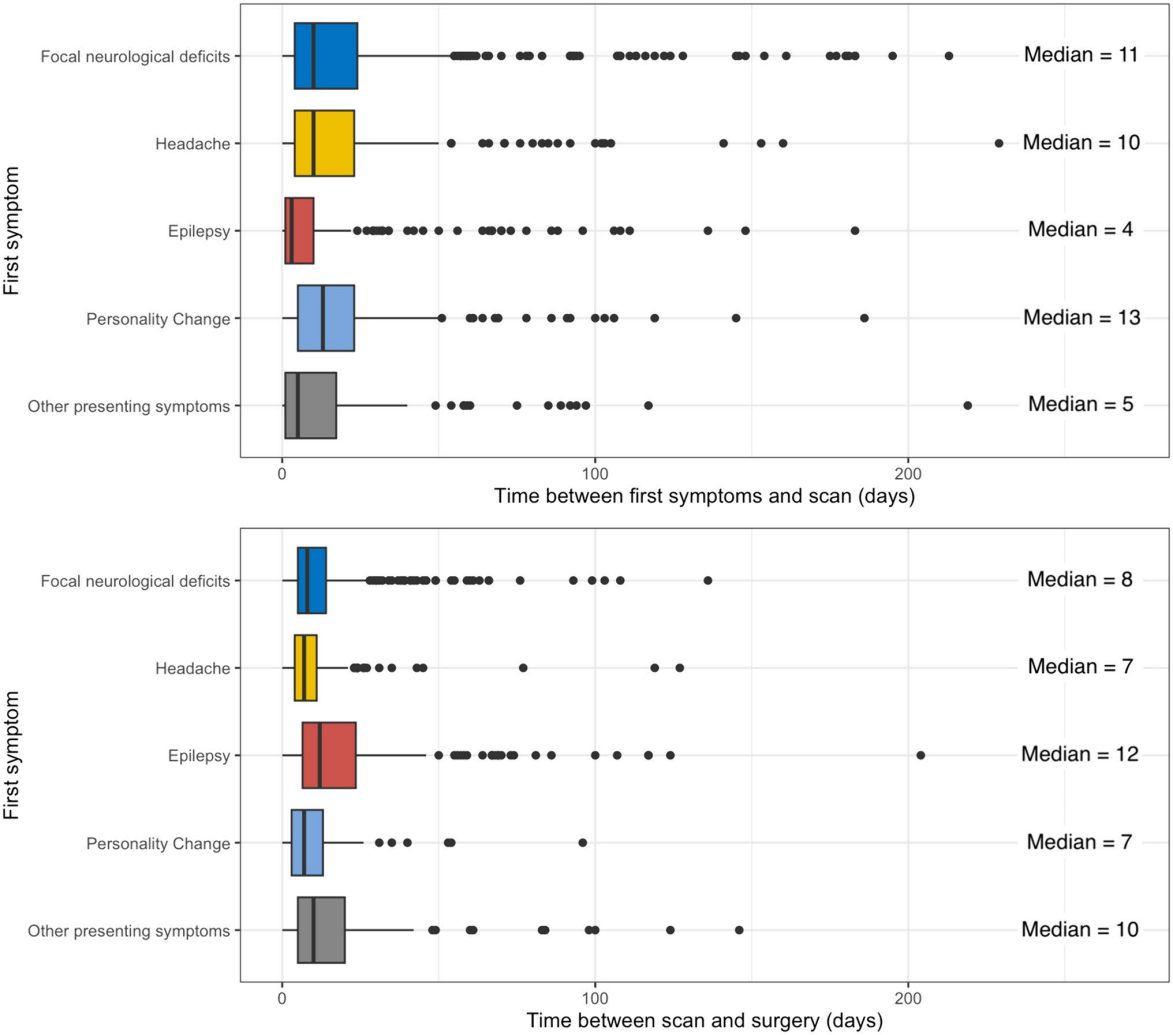
Clinical time intervals. **A** (upper figure) Time between first symptoms and scan. Boxplots show the distribution of time intervals (in days) between different types of first symptoms and first diagnostic scan with MRI. Median values vary between symptom categories (shortest interval in epileptic patients, longest interval in patients with personality changes). **B** (lower figure) Time between scan and surgery. Boxplots as previously described for distribution of time intervals (in days) between first diagnostic scan and first resection. Median values show the shortest interval in patients with personality changes, and the longest interval in epileptic patients (vice versa to A)

In Fig. 2A, timespans from the first presenting symptoms to the first diagnostic imaging (MRI) in days are displayed. Depending on the type of symptom, median time to diagnostic imaging ranged from 4 days in patients with epilepsy to 13 days in patients with personality changes.

Figure 2B shows the time periods from date of first diagnostic imaging to date of first neurosurgical operation. Likewise, timespans differed between presenting symptoms, e.g., 7 days in patients with headache vs. 12 days in case of epileptic seizures.

### Treatment approaches

The initial surgery was either gross total resection (GTR), subtotal resection (STR), or biopsy. The extent of resection (GTR, STR) was rated by the operating neurosurgeon.

GTR was achieved in 579 patients (40.8%), 396 (27.9%) had STR, and 423 (29.8%) underwent a biopsy procedure. In 22 cases (1.5%), the surgical approach is unknown (information not available during data collection). 5-ALA was used in each type of surgery, most commonly in GTR (390, 67.4% of all GTR patients), followed by STR (195, 49.2% of all STR patients) and biopsy (124, 29.3% of all biopsied patients). In 2014, 5-ALA-assisted neurosurgery was applied to 38.3% of all patients, and by 2018 its use had increased to 55.0%.

Most patients started postsurgical treatment approximately one month (median 31 days, 95% confidence interval (CI) 24–44 days) after first neurosurgical procedure. 107 patients (7.5%) could not receive any postsurgical treatment or did not consent to radiotherapy or to chemotherapy. In further 272 patients (19.2%), therapy data were missing due to the following reasons: decease before potential start of post-surgical treatment; patients lost to follow-up; missing data / information on therapy not available during data collection.

**Fig. 3:**
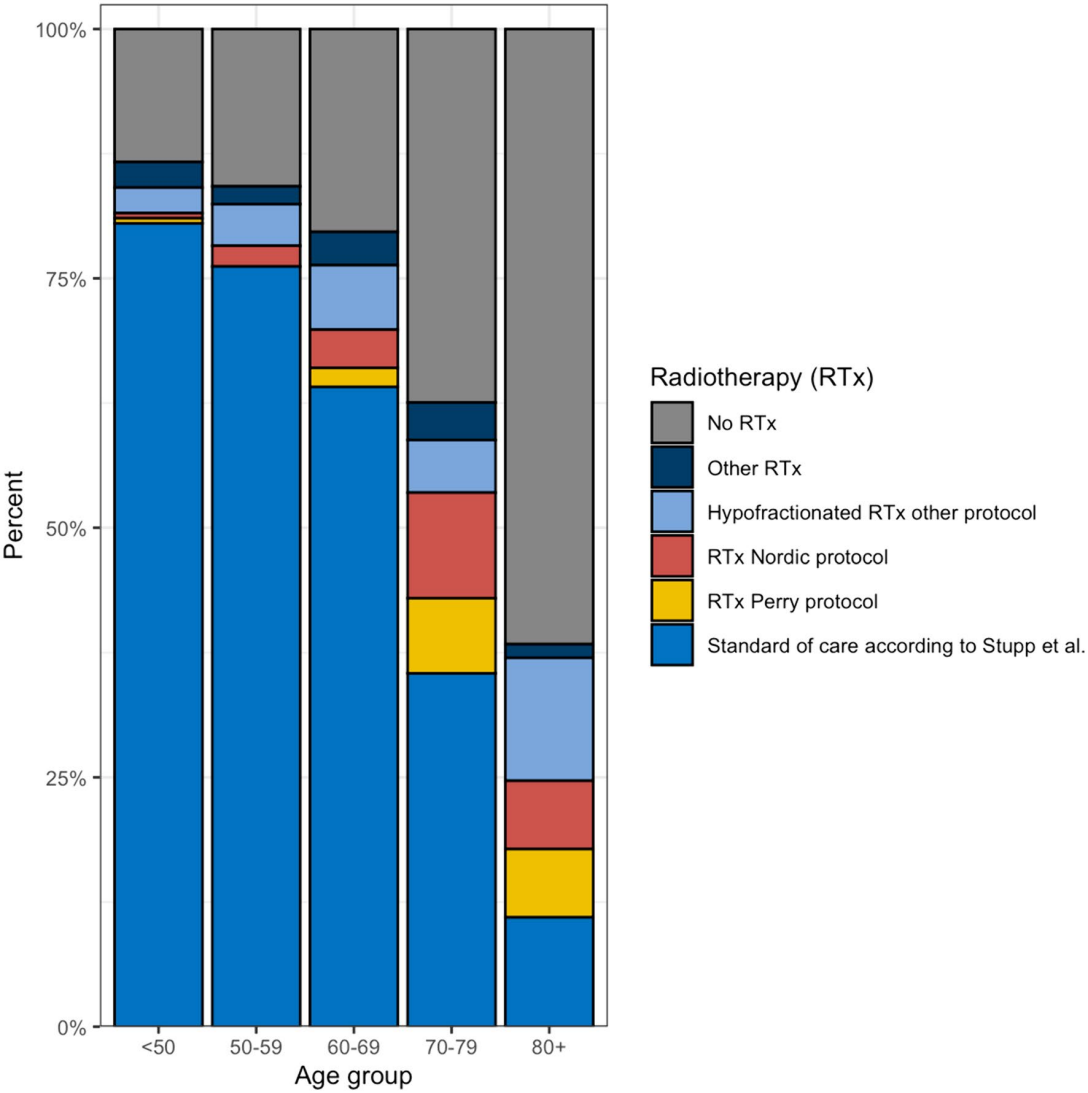
Radio-oncological treatment strategies in relation to patient age. Stacked bar chart showing percentage distribution of radio-oncological therapy regimens in different age groups of Austrian glioblastoma patients. In the younger age groups, relatively high numbers of patients were treated according to standard of care, whereas elderly patients receive more frequently age-adjusted treatment

Of all 1,420 patients, 1,041 (73.3%) started first line post-surgical treatment. 830 (58.5%) started radiochemotherapy following the SOC protocol according to Stupp et al. [4]: 60 Gray (Gy) in 30 fractions of 2 Gy, combined with temozolomide (TMZ). Other radio-oncological treatment strategies included: the Canadian Cancer Trials Group CCTG CE.6 randomized clinical trial protocol designed for elderly glioblastoma patients (N = 44, 3.1%) [23]; the hypofractionated scheme with 34 Gy from the Nordic trial (N = 71, 5.0%) [24]; as well as other individually adjusted radiotherapy protocols (Fig. 3).

Of 830 patients (58.5% of the whole patient cohort), who started radiochemotherapy according to the SOC, completion of treatment was documented in 302 patients (21.3%). The remaining 528 patients (37.2%) could not complete therapy due to progression of disease and/or therapy toxicity. In addition, tumor treating fields (TTF) were applied in 43 patients (3.1%). First-line treatment strategies were similar throughout centers across Austria. Disease progression, as defined by the initiation of second-line treatment, was diagnosed in 691 patients (48.7%). Time to progression was 7.8 months in median (95% CI 6.5–8.0 months).

Treatment upon relapse showed great variability. 170 patients (12.0%) underwent a second surgical procedure, 467 (33.0%) received systemic treatment after progression, and 173 (12.2%) were re-irradiated.

Upon disease progression, 424 (29.9%) received anti-angiogenic therapy.

### Survival analyses

**Fig. 4:**
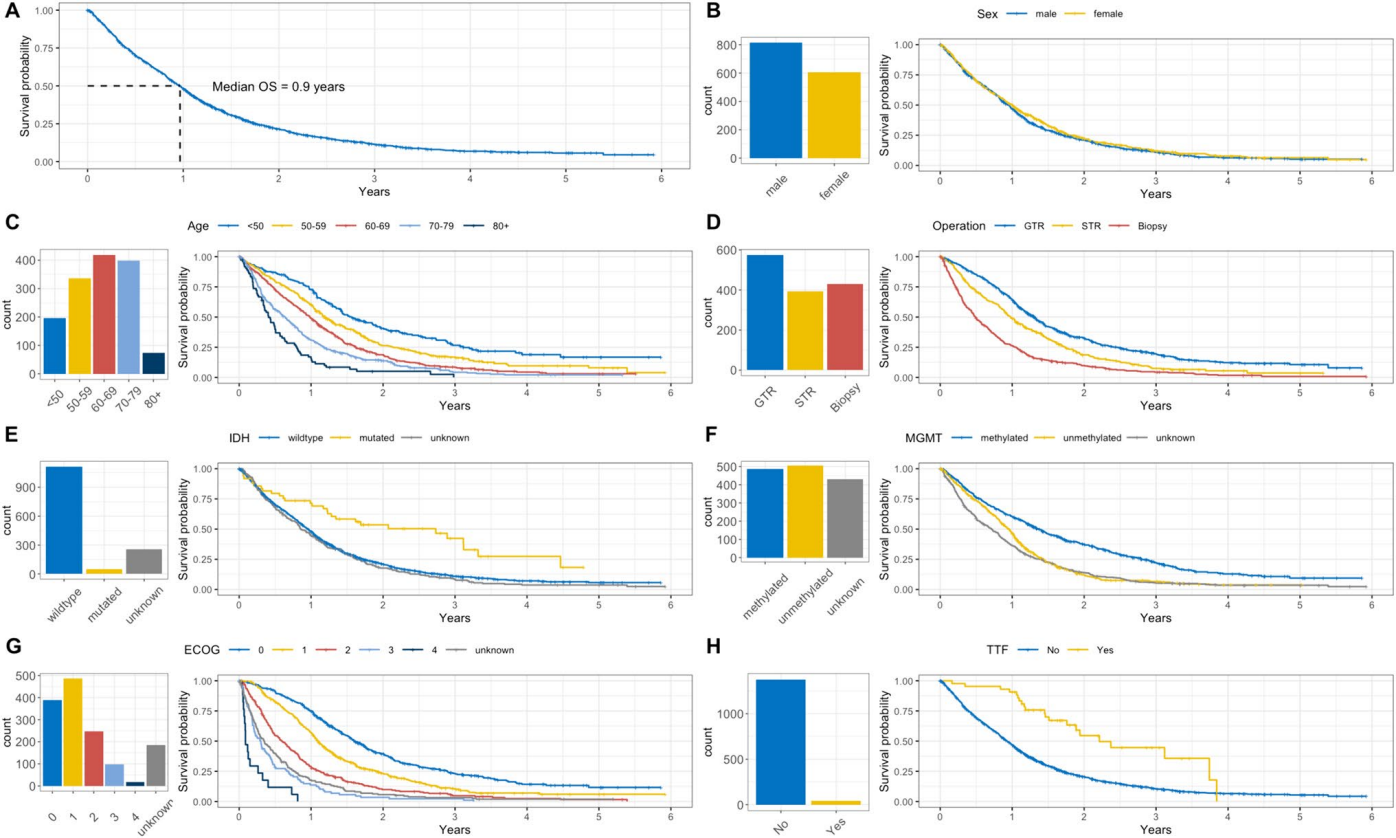
Results of univariate survival analyses. Kaplan–Meier plots showing overall survival **A** as well as analyses of specific prognostic factors (B-H). Kaplan–Meier plots are accompanied by bar charts showing numbers of patients in different categories. **B** Sex: there is no sex difference with regard to outcome. **C** Age: there is a clear indirect correlation: young patients show a significantly more favorable outcome as compared to elderly patients. **D** Extent of resection: there is a direct correlation: patients with gross-total/subtotal resection survive significantly better than patients with biopsy only. **E** IDH-status: significantly better survival in IDH-mutated cases. **F** MGMT-promoter-methylation: significantly better survival in cases with MGMT-promoter methylation. **G** ECOG score: there is a clear indirect correlation: patients with a low postoperative ECOG score survive significantly better as compared to patients with a high ECOG score. **H** Tumor treating fields (TTF) therapy: the small group of patients who received TTF-therapy show a significantly better outcome

Median overall survival (OS) of all glioblastoma patients in this study cohort was 11.6 months (95% CI 10.7–12.3), and 10.9 months (95% CI 10.3–11.8) in the cohort with proven IDH-wildtype (1,115/1,420). Median OS in the patient group ≤ 65 years receiving postoperative SOC therapy according to Stupp et al. [4] was 16.1 months (95% CI 15.5–17.2). In patients older than > 65 years receiving any kind of postoperative therapy, median OS was 11.2 months (95% CI 10.4–12.2). Figure 4 shows survival relative to specific prognostic factors, including sex, patient age, extent of resection, IDH-status, MGMT-promoter-methylation, ECOG score, and TTF therapy. A peculiar finding is the relatively high fraction of patients in whom the MGMT status is unknown. In these cases, explanatory information for the lack of MGMT testing is most often missing, e.g., if testing was not done for particular clinical reasons; or MGMT testing was not available in all centers at the beginning of the registry; or if the amount of biopsy tissue available was not sufficient for testing. Of note, survival in the small cohort of glioblastoma patients with TTF-therapy (N = 43) showed a significantly better outcome than patients without TTF-therapy.

**Fig. 5:**
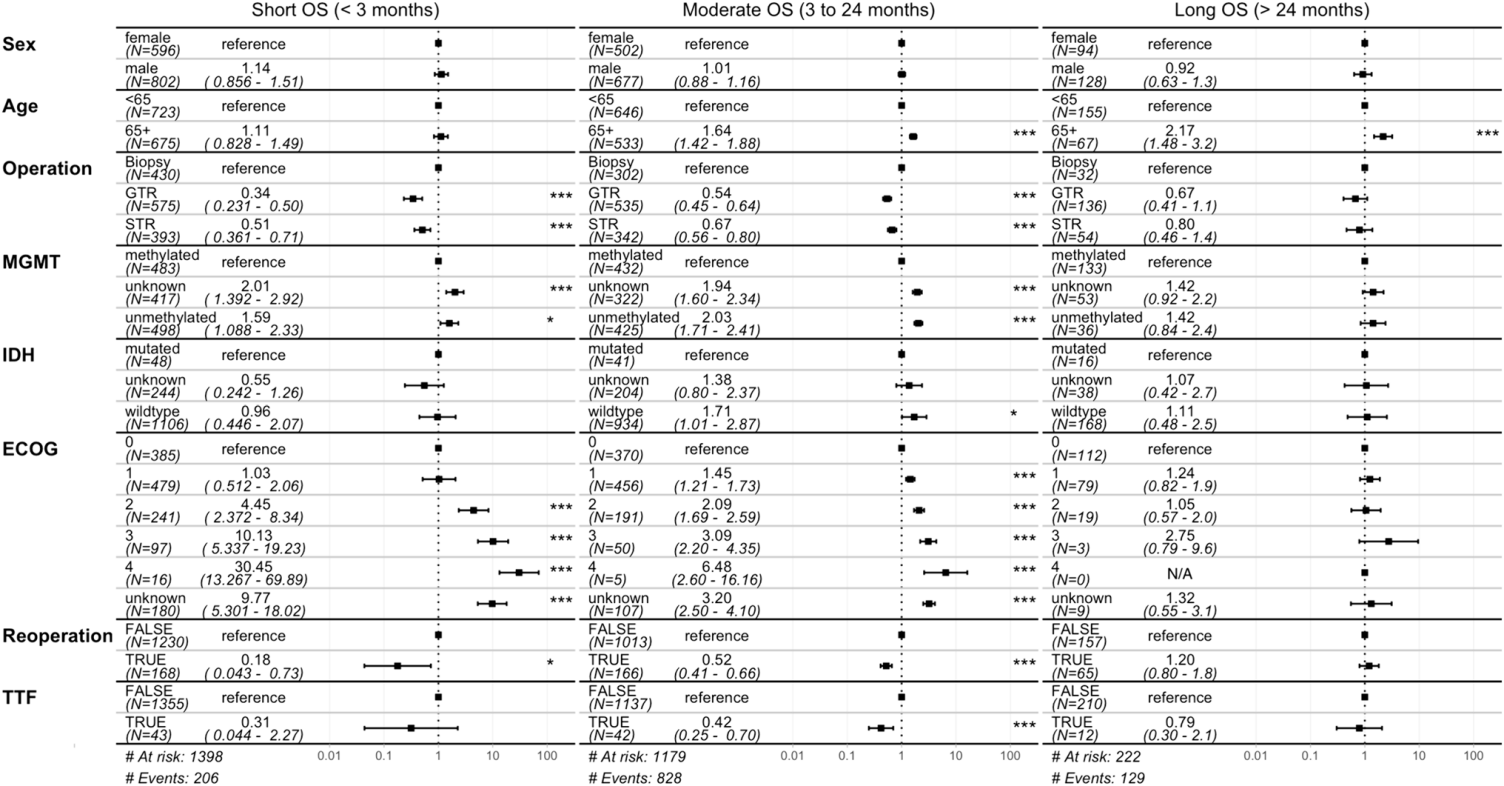
Results of multivariate survival analyses. Forest plots showing results of multivariate survival analyses for specific prognostic factors in three different groups of overall survival (OS): Short OS (< 3 months), Moderate OS (3 to 24 months) and Long OS (> 24 months). Statistically significant values are indicated with one (slight significance) or three asterisks (high significance). In the Short OS group, the strongest independent predictor for survival is the ECOG score. In the Moderate OS group, factors with high independent impact on survival comprise: patient age; extent of resection; MGMT promoter methylation status; ECOG score; reoperation; and use of TTF. In the Long OS group, the single strong independent predictor for survival is patient age

In Fig. 5, multivariate survival analyses using Cox’s regression model are displayed in a forest plot. The total number of analyzed patients is 1,398/1,420 patients, because in 22 patients the extent of resection was unknown, and this small group was excluded to avoid any distortion of results. Analyses are split into three intervals: Short OS (less than 3 months), Moderate OS (3 to 24 month) and Long OS (more than 24 months), in order to learn the relative impact of various patient parameters on outcome over time. The following parameters were analyzed: sex, age, extent of resection, MGMT methylation status, IDH status, postoperative ECOG performance status, re-resection, and TTF.

No statistically significant differences in OS are detected between female and male patients.

Comparison of age (split into two groups, < 65 and ≥ 65 years) shows a statistically significant risk for elderly patients, especially in the moderate and long OS intervals. Further, statistically significant results of hazard ratio in different OS intervals include: beneficial effects of gross total resection (GTR) and subtotal resection (STR) in the short and moderate OS intervals compared to biopsies; unfavorable outcome of patients with unmethylated MGMT promoter status (compared to methylated) in the moderate OS interval; unfavorable patient outcome associated with worsening postoperative ECOG performance score in the short and moderate OS intervals; favorable outcome effects in patients with re-resection compared to those with only one operation in the short and moderate OS intervals; favorable outcome effect in patients with use of TTF in the moderate OS interval (for further details see Fig. 5).

A slight statistically significant difference was found regarding IDH status within the group with moderate overall survival, depicting a higher risk (hazard ratio 1.71, 1.01–2.87) for patients with IDH wildtype compared to IDH mutation.

The maximum follow-up observation period in our study was 6 years, and only the subcohort of 264/1,420 patients included in the first year of data registration (in 2014) can be considered as long-term (≥ 5 years) follow-up group. Long-term survival (≥ 5 years) was observed in 13/264 patients (4.9%). The majority of patients in this small group of long-term survivors belonged to the younger age group (< 65 years: 11/13), usually had gross total tumor resection (10/13), a favorable postoperative ECOG score (ECOG 0–2 in 12/13 cases, in 1/13 unknown), a total tumor resection (10/13), and postoperative standard of care therapy (13/13); 5/13 were female, and 8/13 were male. MGMT promoter was methylated in 7/13, unmethylated in 1/13, and in 5/13, the methylation status was unknown. IDH was wildtype in 8/13 patients, and in 5/13 patients, the IDH status was unknown.

## Discussion

For the first time, we performed a retrospective nation-wide patterns of care and outcome investigation of glioblastoma patients in the Austrian population. The size of the Austrian population is steadily growing and the number of inhabitants has exceeded the mark of 9 million in 2022 [25]. Our study enterprise was accomplished by cooperation of all Austrian neuro-oncology centers. Herein, we provide detailed information on 1,420 identified adult glioblastoma patients initially diagnosed during the time period 2014–2018 in the real-world setting.

Comparable studies used data extracted from, e.g., the French brain tumor data bank [26, 27], Zurich glioblastoma cohort [12] and the National Cancer Database (NCDB) of the United States of America [28]. Our population-based glioblastoma study is unique regarding the spectrum of investigated parameters: it provides data on the prognostic impact of biological tumor characteristics (IDH, MGMT), the neurosurgical use of 5-ALA for intraoperative tumor visualization, and treatment modalities including extent of resection, postsurgical treatment, and treatment at relapse. Our study also provides information on the implementation of hypofractionated radiation in elderly patients as well as complementary therapy approaches such as tumor treating fields (TTF) including related outcome data in the real-world setting.

The robust quality of our dataset is supported by the impact of clinical parameters on outcome—in univariate und multivariate analysis we made the following observations in line with the literature [29]: sex is not related to outcome [10]; there is a better outcome for patients with gross total resection compared to subtotal resection or biopsy as first operation [30–33]; there is also a clear outcome association with patient age, ECOG score, MGMT promoter methylation status [21], IDH-mutation status [16], and adherence to multimodal therapy [10, 29, 34].

The use of 5-aminolevulinic acid (5-ALA) in neurosurgery of glioblastoma has been introduced early in Austria, and is used to optimize maximal safe resection and as an intraoperative marker for identification of CNS tissue areas with tumor cell infiltration during biopsy [17, 33]. Our data show that 5-ALA application has translated into common clinical practice: in the majority of cases, gross total and subtotal resections were performed with the help of 5-ALA; in addition, 5-ALA was also used in biopsies for intraoperative detection of pathological tissue in a considerable proportion of cases.

A finding of interest in our study is the association of TTF application with prolonged survival both in univariate as well as in multivariate survival analysis. TTF is increasingly applied as a standard of care intervention in glioblastoma [35, 36]. Its effect on outcome has so far been reported in controlled prospective therapy trials [6]. Herein, we present evidence of an association with prolonged survival in a population-based investigation, although the TTF subgroup is rather small as compared to the total study cohort. The low patient numbers in the TTF subgroup can be explained by the retrospective nature of our study, with case collection dating back until 2014 (early implementation phase of TTF in Austria). Follow-up studies investigating the use of TTF in a larger patient cohort with detailed usage parameters will help to identify any potential bias and objectify the independent effect of TTF application on patient outcome in the real-world setting more precisely.

Data and results of our study further indicate that efforts were made in our Austrian neuro-oncology units to consider the individual patient condition and therapy tolerance before choosing the appropriate postsurgical treatment options for glioblastoma. A fraction of elderly patients was treated according to protocols for younger patients and vice versa some of the younger patients received therapy regimens established for elderly patients. In the patient group up to 65 years of age, the current SOC [4] was initiated in the majority of the patients. Their survival was 16.1 months in median, which is comparable to that of whole patient cohorts included in study protocols [6].

In line with previous reports, first-line therapies in our study cohort were relatively homogenous following established age-adjusted treatment protocols, whereas in case of disease progression treatment was more individualized. Of note, bevacizumab—although not approved as standard therapy of glioblastoma by the Austrian health authorities—was frequently applied in the second line systemic treatment setting, mostly with the aim to alleviate symptoms of tumor-related brain edema and to support palliative and comfort care.

In our study, analysis of long-term survival is limited since the observation period was 6 years at maximum. Nevertheless, we observe that our study population includes patients surviving more than 5 years (N = 13/264 = 4.9%). Most of these patients were younger than 65 years of age (N = 11, 84.6%) and had gross-total or subtotal resection, but occasionally, also elderly patients had long-term survival. However, all of those long-term survivors had a favorable postoperative ECOG score (0 to 2); MGMT promoter was methylated or unmethylated in the long-term survivors. These findings in our small group of long-term survivors are basically in line with the recently reported findings in the EORTC 1419 ETERNITY study of long-term surviving glioblastoma patients [37].

## Conclusions

We conclude that the analysis of ABTR data can provide a sound basis for benchmarking and quality control in the Austrian real-life setting of glioblastoma care and outcome. Beyond Austria, the aggregated data allow international comparison with other national registries.

In the future, continued data collection and analysis will be based on glioblastoma definition according to the most recent fifth edition of the WHO classification released in 2021, which also includes the concept of molecular glioblastoma, and does not include high-grade IDH-mutant astrocytic gliomas anymore [19].

Precision medicine approaches are currently gradually implemented for eligible glioblastoma patients. It will be of interest to measure the effects of these new therapeutic interventions at the population level in the future.

Limitations of our study: Due to the retrospective nature of the study (1) clinical and molecular data could not be fully assessed in all patients; (2) additional data of interest, e.g., neurocognitive testing or more detailed intraoperative information remain to be collected and analyzed in future more recent patient cohorts or prospective studies which will also allow the use of the most recent CNS WHO 2021 brain tumor classification [19]; (3) outcome follow-up was lost in some patients because of migration; (4) long-term follow-up ≥ 5 years is limited to a comparatively small fraction of the total patient cohort.

## Data Availability

The datasets generated during and/or analyzed during the current study are stored in the ABTR-SANOnet database at the Medical University of Vienna. Access to data can be obtained on reasonable request via the ABTR-SANOnet coordinators Johannes A. Hainfellner and Stefan Oberndorfer.
